# Acceptance of a digital therapy recommender system for psoriasis

**DOI:** 10.1186/s12911-023-02246-9

**Published:** 2023-08-04

**Authors:** Lisa Graf, Falko Tesch, Felix Gräßer, Lorenz Harst, Doreen Siegels, Jochen Schmitt, Susanne Abraham

**Affiliations:** 1 Center for Evidence-Based Healthcare, University Hospital Carl Gustav Carus and Faculty of Medicine Carl Gustav Carus, TU Dresden, Dresden, Germany; 2 Institute of Biomedical Engineering, TU Dresden, Dresden, Germany; 3 Center of Evidence-Based Healthcare, Branch Office at the Medical Campus Chemnitz, University Hospital Carl Gustav Carus and Faculty of Medicine Carl Gustav Carus, TU Dresden, Dresden, Germany; 4 Department of Dermatology, Faculty of Medicine Carl Gustav Carus, University Hospital Carl Gustav Carus, TU Dresden, 01307 Dresden, Germany

**Keywords:** Psoriasis, Therapy decision, Clinical decision support system (CDSS), Survey

## Abstract

**Background:**

About 2% of the German population are affected by psoriasis. A growing number of cost-intensive systemic treatments are available. Surveys have shown high proportions of patients with moderate to severe psoriasis are not adequately treated despite a high disease burden. Digital therapy recommendation systems (TRS) may help implement guideline-based treatment. However, little is known about the acceptance of such clinical decision support systems (CDSSs). Therefore, the aim of the study was to access the acceptance of a prototypical TRS demonstrator.

**Methods:**

Three scenarios (potential test patients with psoriasis but different sociodemographic and clinical characteristics, previous treatments, desire to have children, and multiple comorbidities) were designed in the demonstrator. The TRS demonstrator and test patients were presented to a random sample of 76 dermatologists attending a national dermatology conference in a cross-sectional face-to-face survey with case vignettes. The dermatologist were asked to rate the demonstrator by system usability scale (SUS), whether they would use it for certain patients populations and barriers of usage. Reasons for potential usage of the TRS demonstrator were tested via a Poisson regression with robust standard errors.

**Results:**

Acceptance of the TRS was highest for patients eligible for systemic therapy (82%). 50% of participants accepted the system for patients with additional comorbidities and 43% for patients with special subtypes of psoriasis. Dermatologists in the outpatient sector or with many patients per week were less willing to use the TRS for patients with special psoriasis-subtypes. Dermatologists rated the demonstrator as acceptable with an mean SUS of 76.8. Participants whose SUS was 10 points above average were 27% more likely to use TRS for special psoriasis-subtypes. The main barrier in using the TRS was time demand (47.4%). Participants who perceived time as an obstacle were 22.3% less willing to use TRS with systemic therapy patients. 27.6% of physicians stated that they did not understand exactly how the recommendation was generated by the TRS, with no effect on the preparedness to use the system.

**Conclusion:**

The considerably high acceptance and the preparedness to use the psoriasis CDSS suggests that a TRS appears to be implementable in routine healthcare and may improve clinical care. Main barrier is the additional time demand posed on dermatologists in a busy clinical setting. Therefore, it will be a major challenge to identify a limited set of variables that still allows a valid recommendation with precise prediction of the patient-individual benefits and harms.

**Supplementary Information:**

The online version contains supplementary material available at 10.1186/s12911-023-02246-9.

## Background

### Clinical decision support Systems

So-called Clinical Decision Support Systems (CDSS) are intended to assist decision making in difficult clinical situations and to help implement guideline-based treatment [1]. However, computer-based CDSSs traditionally rely on a well-tended knowledge base and therefore react poorly to missing or heterogeneous data [2]. Furthermore, the inference or reasoning mechanism, i.e. the algorithms which lead to a decision support, are often non-transparent black boxes [3]. With these shortcomings in mind, recommender systems known from E-commerce, such as the suggestions made by Netflix on what to watch next, gain interest in the medical community without being used comprehensively [2]. The basic principle of such recommender systems is that they suggest treatment options by comparing patients based on information provided in Electronic Health Records (EHRs) [4]. The barrier of lacking transparency of CDSSs, as concluded by Khairat et al. and Shortliffe et al., may be overcome by such a similarity-based recommender [1, 5].

Due to their limited use to date, there are no relevant studies on the acceptance of recommender systems among physicians so far, especially in a field such as dermatology.

Therefore, this research aims at determining whether dermatologists are willing to use a therapy recommendation system in general and to specify predictors relevant for the acceptance of a digital recommendation system for the treatment of psoriasis.

### Burden of Psoriasis

The prevalence of psoriasis in the German population is approximately 2% [6]. As psoriasis not only affects the skin but comes with several comorbidities, such as cardiovascular diseases, arthritis and depression [7–9], topical treatment of the skin lesions (plaques) for some patients is not enough. Therefore, numerous European guidelines suggest systemic treatments with focus on reducing the risk of chronic inflammatory conditions, such as cardiovascular events [10, 11]. Due to the high burden of patients suffering from psoriasis, in the year 2014 the World Assembly of the World Health Organization (WHO) passed a resolution on psoriasis to raise awareness regarding the disease and to improve the health care of individuals with psoriasis [6].

### Dermatologist prescription behavior

The German guideline advices the use of systemic therapy in all cases of moderate to severe psoriasis [10]. Despite the growing number of well-tolerated and effective drugs, a high undertreatment of patients suffering from psoriasis can be assumed. In a recent survey of psoriasis patients, 45.9% of participants were not receiving medical care at the time of the evaluation. Of the remaining 54.1%, nearly 60% were not satisfied with their current treatment [12]. In a further evaluation of patients with moderate to severe psoriasis only about 15% were treated with biologics [13]. Uncertainty regarding the administration of systemic therapy and monitoring of its course could contribute to adverse effects of systemic therapy, such as severe infections [14, 15].

The Multinational Assessment of Psoriasis and Psoriatic Arthritis (MAPP) survey (North America and Europe) also indicated an underuse of systemic therapy in patients with psoriasis. The survey included 391 dermatologist in North America and Europe [16]. In this study, 53.5% of dermatologists reported using topical treatment as monotherapy for moderate to severe psoriasis. Concerns about the long-term safety, tolerability and efficacy of therapy were the reasons for underuse, non-initiation or continuation of systemic therapy. 60% of the dermatologists stated that they require more time in dermatological practice for the treatment of psoriasis patients than for patients with other diseases [16]. In addition, the results of the NPF (National Psoriasis Foundation) surveys, which were conducted from 2003 to 2011 among patients in the USA with psoriasis and psoriatic arthritis indicate an underuse of systemic therapy [17]. The complexity of psoriasis treatment was further increased by common comorbidities.

López Estebaranza et al. undertook a mail survey of Spanish hospital dermatologists about the treatment of patients with moderate to severe psoriasis. Biologics were the most frequently used monotherapy in this setting. But nearly two thirds of dermatologists stated that they would initially strive for a conventional systemic therapy and only switch to a biological therapy after 1 to 2 years, mostly due to insufficient control of disease activity [18]. Due to the large number of possible high-priced therapy options combined with high time pressure in dermatological practices, it is increasingly difficult to determine the optimal therapy option.

## Methods

To evaluate the need of TRSs, a prototypical demonstrator was presented to dermatologists in a cross-sectional face-to-face survey with case vignettes.

### Demonstration of the digital recommender system

**Fig. 1:**
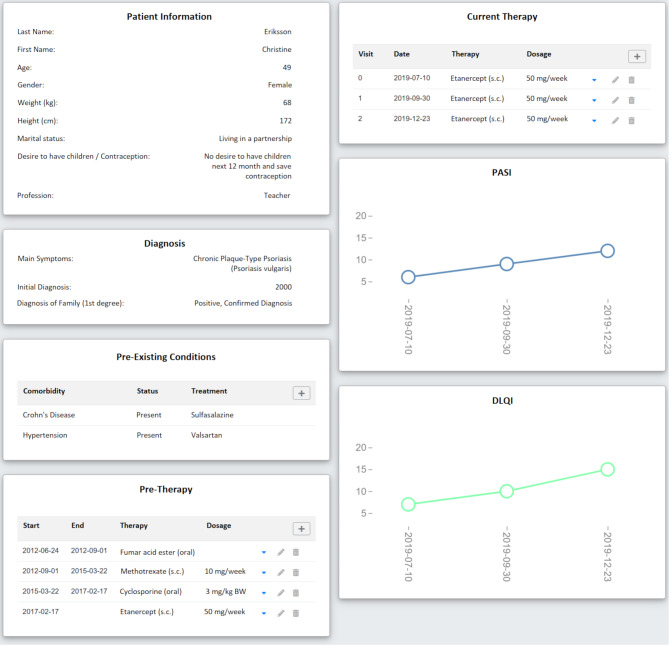
Exemplary medical history for patient 1 with psoriasis vulgaris and Crohn’s disease

Three different psoriasis cases were developed on the basis of real patients at the Dresden Dermatological Clinic. Each patient’s characteristics were adapted to represent a specific clinical scenario. The modified characteristics included medical history, features such as name, age, sex, and weight, as well as year of first diagnosis, family history and wish to have children. Comorbidities, comedications, premedication, current medication, and reasons for therapy changes, such as increase in transaminases or creatinine, were modified, and added to the prototypical cases. The duration and dosages were broken down transparently, so that the dermatologist interviewed had a conclusive picture and the necessity of a change in therapy within in the prototypical cases become comprehensible. One of the cases can be seen in Fig. 1.

**Fig. 2:**
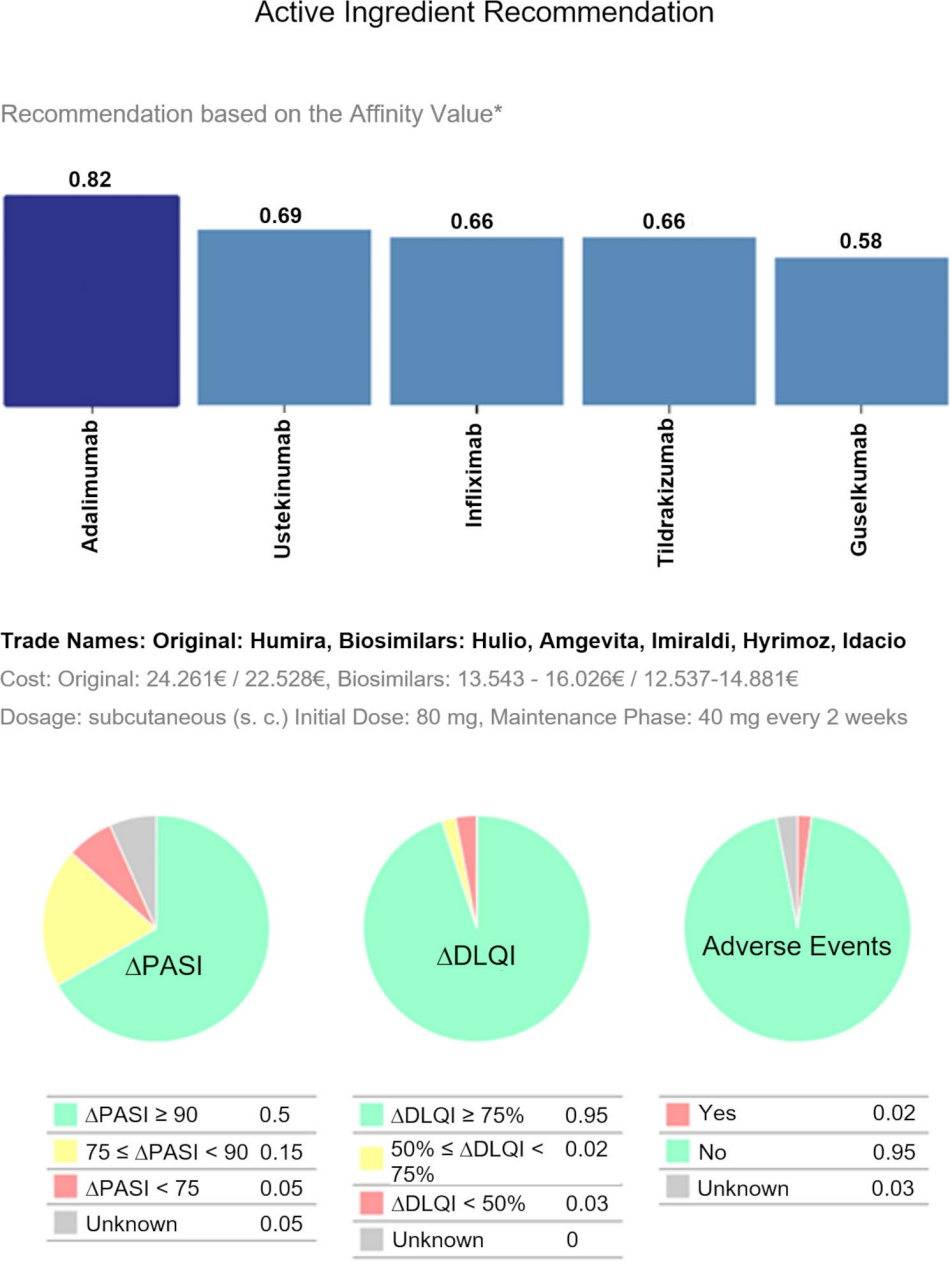
Example of a therapy recommendation for patient 1 with Crohn’s disease as comorbidity, * Affinity Value: summary measure of frequency, improvement in DLQI score (Δ DLQI), improvement in PASI score (ΔPASI), and occurrence of adverse events for each treatment option. Therapy recommendation based on most similar patients

The prototypical CDSS provides a recommendation for each of the three cases based on the current S3 guideline [10], professional information including various drug effectiveness and safety studies and the experience of the collaborating dermatologists (University Hospital Dresden). Furthermore, Patient Reported Outcomes (PROMs) are factored into the therapy recommendations. Figure 2 shows an exemplary therapy recommendation output. In addition, the dermatologists were provided with further information about the therapy recommendation (see Fig. 2), which is supposed to support them in their decision making. These included the therapy interval, therapy dosage and the costs in the first or following years, based on current price lists.

The following briefly describes the three case studies. Each of the three exemplary patients had already received systemic therapy:

- Patient with psoriasis vulgaris and inflammatory bowel disease (Crohn’s disease).
- Patient with psoriasis vulgaris and psoriasis guttata under methotrexate (MTX) therapy with newly wish to have children.
- Patient with psoriasis vulgaris with nail involvement and many comorbidities and secondary treatment failure of the TNF-alpha-antibody etanercept.

Comorbidities, time course, previous and current systemic therapies, and treatment response are described. The upper line chart shows the trend of the psoriasis area and severity index (PASI) over the last 3 visits, while the lower line graph illustrates the trend of Dermatology Quality of Life Index (DLQI) during the same period. As PASI and DLQI have increased, a therapy change is indicated.

### Questionnaire and participants recruitment

The questionnaire consisted of 30 items (see appendix). Usability of the system was operationalized by using the System Usability Scale (SUS), which has been tested and validated for several languages, including German [19]. To further assess the usability of the demonstrator, items called “Lack of confidence in correct operation” and “Designations not intuitively understandable” were added. The former also breaches the subject of experience with technology [20]. Perceived usefulness was translated into the need to apply a recommender system, which is bound to be less pronounced for physicians with more job experience. Also, an item called “No added value to the previous way of working” was added. Effort expectancy was operationalized using the item “Too much time required”. To capture the importance of transparency within the recommender system, the following items were added: “Unclear how recommendation comes about” and “Relevant information missing”. The acceptance was asked for patient eligible for systemic therapies, patient with comorbidities or pregnancy and for patients with special subtypes of psoriasis.

The cross-sectional survey of 76 face-to-face interviews with German dermatologist was undertaken in February 2020. 67 of the interviews were conducted at the annual congress of the German dermatology society in Dresden by four different interviewers, the others at the clinic for Dermatology at the university hospital Carl Gustav Carus at TU Dresden. No physician registry or incentives were used to recruit participants.

All interviews were conducted with tablet computers. Study data were collected and managed using REDCap electronic data capture tools hosted at the university hospital Carl Gustav Carus at TU Dresden. The dermatologists were first familiarized with the recommendation system by means of one of the three case studies. Afterwards, the dermatologists had time to look at the demonstrator and try it out for themselves. The time needed for both was about 5 to 10 min. The dermatologists were allowed to ask questions at any time. The function of the demonstrator was limited, so that the dermatologists could not change the case by input. However, the treatment recommendation could be changed depending on the focus of the objective, improvement of PASI, DLQI and avoidance of adverse events (Fig. 2). Dermatologists were next asked to complete a questionnaire at the tablet. The questionnaire was the same for all three case studies. The time needed to complete the questionnaire was about 10–12 min.

### Statistical analysis

For descriptive data analysis, frequency tables and kernel density plots were used. Three Poisson regression models were estimated for three configurations of the system: either for patients eligible for systemic therapies, patient with comorbidities or pregnancy or for patients with special psoriasis forms. The estimates were displayed as risk ratios with a 95% confidence interval. A value above 1 shows an increased risk ratio and a value below 1 a decreased risk ratio. Due to the binary outcome robust standard errors were applied [19]. The software R Version 3.6.2 was used for data analyses [21]. For raw data see appendix “Dataset_BMC”.

## Results

Characteristics of participants.

Table 1 describes the characteristics of the survey participants. The participating dermatologists were aged between 27 and 78 years (average age 40.9 years) and had an average professional experience of 12.75 years. However, the question of professional experience was only answered by 67 of 76 participants. The proportion of dermatologists still in training was high, especially among hospital dermatologist: 31 out of 39 hospital dermatologists were still in training. Significantly more women than men participated in the survey (76%). Almost half (48%) of the dermatologists were working on an outpatient basis. Dermatologists were asked to evaluate the user-friendliness of the therapy recommendation system based on the SUS. Scores between 37.5 and 100 were achieved. A score higher than 68 means that the system has acceptable usability [20]. Accordingly, the usability of recommendation system was rated as acceptable with an mean SUS of 76.8. Cronbach’s Alpha for SUS scale in this population is acceptable (0.796), yet lower than in previous studies using the scale [21].

**Table 1:** Characteristics of interviewed dermatologists and their assessment of the recommender

Characteristic	Dermatologist	n
Age (mean/range)	40.9 (27–78)	72
Female (n/percent)	58 (76.3)	76
Job experience in years (mean/range)	12.75 (1–55)	67
Outpatient treatment (n/percent)	36 (48)	75
Number of patients per week (n/5 and more in percent)	47 (62.7)	75
Usability of the recommender (mean/range)	76.84 (37.5–100)	72
Acceptance for patients eligible for systemic therapies (n/percent)	62 (81.6)	76
Acceptance for psoriasis subtypes (n/percent)	33 (43.4)	76
Acceptance for comorbidities (n/percent)	38 (50.0)	76

62.7% of dermatologists stated that they treat at least 5 patients with psoriasis per week. In addition, the dermatologists were asked in which cases they would use the therapy recommendation system. The highest level of acceptance (82%) was achieved for a usage in treatment of patients eligible for systemic therapy, while 50% of the participants would use it for the treatment of patients with comorbidities. However, only 43% of the participants stated to be willing to use the system for patients with special subtypes of psoriasis (Table 1).

The answers were independent of which of the three pseudo patients was shown to the participants.

No differences were found in the SUS values according to the presented case and the dermatologists’ age and years of job experience.

There was a small sex difference between dermatologists concerning the acceptance of the system for the treatment of patients who are eligible for systemic therapy (male: 82.7% vs. female: 77.8%). There were large differences in the acceptance of the software for the treatment of patients with comorbidities or pregnant patients (58.6% vs. 22.2%) and patient with special forms of psoriasis (48.3% vs. 27.8%) in favor of female dermatologists.

### Barriers for the intended use of the recommender system

An additional aim of the survey was to identify barriers which hinder the application of such TRSs in practice. The results are shown in Table 2. The most common reason given by 36 (47.4%) dermatologists was that using the recommendation system was too time-consuming. 21 (27.6%) dermatologists stated that they did not understand how the treatment recommendation system worked and 11 (14.5%) reported a lack of confidence into the system and its reliability to work properly. 7 (9.2%) dermatologists did not see any added value in using the system compared to their conventional way of working. 5 (6.6%) dermatologists thought that relevant information was not included in the system and 3 (4%) respondents felt that the system was not intuitive (Table 2).

**Table 2:** Barriers in usage of the recommender system

Barriers	Amount	Percent
Too much time required	36	47.4
Unclear how recommendation comes about	21	27.6
Lack of confidence in correct operation	11	14.5
No added value to the previous way of working	7	9.2
Others	7	9.2
Relevant information missing	5	6.6
Designations not intuitively understandable	3	4.0

The main barrier to applying the TRS in practice was the additional time required when using the system. Therefore, the dermatologists were divided into 2 groups depending on whether they found the TRS too time-consuming or not. Of the dermatologists for whom an SUS score could be determined, 38 did not consider time to be a barrier, while 34 felt that using the therapy recommendation system would take too much time. The corresponding mean values for SUS were 77.56 and 76.02. Consequently, people who did not chose time as a barrier assessed the therapy recommendation system to be 1.5 points more usable.

Dermatologists who scored at least 10 points above the mean SUS score of 76.84 were 27% (CI: 2.8 to 57.9%) more likely to accept the treatment recommendation system for specific subtypes of psoriasis. Although not significant, female dermatologists might be more inclined to use the therapy recommendation system also for patients with comorbidities or special subtypes of psoriasis compared to their male colleagues (see Table 3). Participants who found the system too time-consuming were 22.3% less inclined to use the recommendation system for patients eligible for systemic therapy (CI: 4.3 to 36.9%). For all three acceptance categories it was shown that dermatologists would use the system regardless of their understanding on how the system generates the therapy recommendation. The age of the participants was not significant in any of the models and was therefore excluded.

**Table 3:** Variables influencing the three acceptance categories. The categories are special subtypes of psoriasis, patients with comorbidities and patients eligible for systemic therapy. Poisson regression for three different acceptance categories. Displayed are relative risks (RR) with a 95% confidence interval (CI). n = 71

Variable	Model acceptance for patients eligible for systemic therapies		Model acceptance for comorbidities		Model acceptance for special psoriasis forms	
	RR	95% CI	RR	95% CI	RR	95% CI
System Usability Scale (SUS) per 10 points	1.093	0.983–1.215	1.198	0.998–1.439	**1.274**	**1.028–1.579**
Female (Ref. Male)	0.922	0.715–1.189	2.213	0.881–5.559	1.711	0.713–4.108
Barrier Too much time required	**0.777**	**0.631–0.957**	0.966	0.637–1.465	0.836	0.500-1.397
Barrier Unclear how recommendation comes about	1.113	0.943–1.31	1.099	0.685–1.762	1.107	0.629–1.949
Outpatient Sector (Ref. hospital)	0.941	0.749–1.182	0.976	0.524–1.816	0.511	0.230–1.139
5 and more patient with psoriasis per week (Ref. up to 4)	0.861	0.683–1.085	0.940	0.522–1.690	0.573	0.267–1.234

### Sensitivity analysis

Restricting the population to those 53 dermatologists with at least an SUS of 68 or higher shows stronger effect estimates for the acceptance of the system for the treatment of special psoriasis subtypes. SUS increased to a risk ratio of 1.426 (CI 0.987–2.061), working in the outpatient sector achieved a risk ratio of 0.368 (CI: 0.146–0.930) and being a dermatologist with five and more patients per weeks achieved a risk ratio of 0.402 (CI: 0.164–0.984).

## Discussion

The results show that the acceptance of a recommender system suggesting treatment options for psoriasis patients was high overall, yet highest for the usage in treatment of patients eligible for systemic therapy. This shows once more the physicians’ need for decision support when faced with the option to administer such treatments [15]. However, in the regression model, no significant association between patient type and acceptance of the system was found, suggesting that the participants of the survey evaluated the system’s perceived helpfulness in general. Only a small fraction of the dermatologists found no added value in the system compared to how they treat psoriasis patients currently. This means that the perceived usefulness of the system is high, making acceptance of the system by dermatologists likely [20]. However, as medical care often takes place under time constrains, a recommender system must not be time-consuming, which, according to the participants, is not achieved with the system studied. Especially in case of systemic treatment, “too much time required” is a significant barrier, showing that the system needs to be improved in this respect. One way to do so could be allowing for automated input of data from clinical information systems of personal health records into the recommendation system.

In addition, dermatologists with many psoriasis patients per week were less willing to use the TRS. A possible reason for this is the higher time pressure due to a high number of psoriasis patients. On the other hand, it can be assumed that they have a better routine in the use of systemic therapy. Combined with the anticipated clinically relevant effect of usability, despite the fact that most of our results were not significant in the first two cases due to small sample sizes, these results show how important ease of use could be for each digital system used by physicians [21]. Usability is not a significant predictor when the system is used to recommend systemic therapy. This fact can be interpreted as another indicator that a recommender system is highly needed, i.e. core readiness is observable in the dermatologist community [22]. The high representation of women in the survey is consistent with the predominance of women in dermatology in general [23]. An above-average number of assistant doctors were among the congress dermatologists. Therefore, the average age was comparatively low.

All in all, the results presented fit into research both on acceptance of CDSSs and telemedicine applications in general: CDSSs are assumed to be better accepted when they fit the physician’s clinical reasoning processes and into the clinical workflow [5]. Moreover, as with every digital health application, usability is of high importance [1]. Research on telemedicine acceptance in general has shown that what matters most for physicians are perceived benefits [20], and the amount of effort they are willing to expend to achieve a result. Surveys to date have shown an inadequate treatment of patients with moderate to severe psoriasis [12, 13, 16, 17]. There were several reasons for this. The main reason was that many new biologics were introduced to the market within a short period of time. Dermatologists were challenged by the rapidly increasing number of treatment options and by different contraindications for concomitant diseases. In addition, therapy costs were rising, particularly due to biologics. However, the increase in costs could be justified by a better treatment response and a reduction in follow-up costs such as occupational disability [24, 25]. An optimally adapted treatment supported by a TRS could thus also save resources (fewer consultations in a special outpatient clinic) and costs. For these reasons, the use of a TRS is a reasonable option to respond to these challenges.

### Limitations

In the survey by J. L. López-Estebaranz et al. [18], 75 dermatologists in the hospital environment were interviewed who were considered to be proven experts in the treatment of psoriasis. In contrast, most of the participants in this study were recruited at a national congress of the DDG (German Dermatological Society). Therefore, most of the survey only includes dermatologists who participate in congresses and therefore show a general interest in research within their field. Furthermore, as the participation was optional, it can be assumed that the participants have an increased tendency to participate in surveys or deem the issue of decision support in psoriasis treatment important.

Finally, the presented research only informs on dermatologists’ intended use of a therapy recommendation system. Whether the results, especially the predictors for acceptance, hold will have to be shown by future research once the system is operational.

## Conclusions

Several surveys found an inadequate treatment of patients with moderate to severe psoriasis. In fact, many new biologics have come on the market within a short period of time. Although they promise good control of psoriasis, the uncertainty due to the high costs and the many new treatment options as well as the contraindications to be considered make their use difficult. Most dermatologists (mean SUS of 76.8) rated the recommendation system proposed to assist with the usage decision as user-friendly. The main barrier to the intention to use the recommendation system was the time it takes to operate the system. The system’s acceptance among dermatologists from the outpatient sector was small when treating patients with special subtypes of psoriasis. A quarter of the participants stated that they did not understand exactly how the therapy recommendation system works. However, this had no influence on the willingness to use the therapy recommendation system. The potential improvement of patient care must be evaluated by means of a randomized controlled study. Dermatologists’ performance with and without the assistance of a TRS must be compared.

### Electronic supplementary material

Below is the link to the electronic supplementary material.

Supplementary Material 1

Supplementary Material 2

## Data Availability

All data generated or analysed during this study are included in this published article [and its supplementary information files, see appendix: “Dataset_BMC”].

## List of Abbreviations

CDSS: Clinical decision support systems
CI: Confidence interval
DDG: Deutsche Dermatologische Gesellschaft
DLQI: Dermatology Quality of Life Index
EHRs: Electronic Health Records
MAPP: Multinational Assessment of Psoriasis and Psoriatic Arthritis
MTX: Methotrexate
NPF: National Psoriasis Foundation
PASI: Psoriasis area and severity index
RR: Relative risk
SUS: System usability scale
TNF: Tumour necrosis factor
TRS: Therapy recommendation systems
WHO: World Health Organisation
