# Supplementary material for: Acceptance of a digital therapy recommender system for psoriasis
Source: BMC Med Inform Decis Mak. 2023 Aug 4;23:150. doi: 10.1186/s12911-023-02246-9 (PMC10401871; doi:10.1186/s12911-023-02246-9)
Supplement: Supplementary file 1 — Supplementary Material 1 [file 12911_2023_2246_MOESM1_ESM.pdf]

In this survey we ask you about the prototype of a therapy recommendation system for psoriasis that you have just tested. In the context of a PhD project, we would like to receive your assessment of it.

Your data will be pseudonymized and cannot be traced back to you.

The contact person is Prof. Jochen Schmitt from the Center for Evidence-Based Health Care at the TU Dresden.

Which patient did you look at?

◯ Christine Eriksson ◯ Katja Müller ◯ Uwe Ostermann

Usability

	Strongly agree	I rather agree	part-part	Rather not agree	Strongly disagree
I think that I would like to use this system frequently.	◯	◯	◯	◯	◯
I found the system unnecessarily complex	◯	◯	◯	◯	◯
I thought the system was easy to use.	◯	◯	◯	◯	◯
I think that I would need the support of a technical person to be able to use this system.	◯	◯	◯	◯	◯
I found the various functions in this system were well integrated.	◯	◯	◯	◯	◯
I thought there was too much inconsistency in this system.	◯	◯	◯	◯	◯
I would imagine that most people would learn to use this system very quickly.	◯	◯	◯	◯	◯
I found the system very cumbersome to use.	◯	◯	◯	◯	◯
I felt very confident using the system.	◯	◯	◯	◯	◯
I needed to learn a lot of things before I could get going with this system.	◯	◯	◯	◯	◯

Acceptance

	Strongly agree	I rather agree	part-part	Rather not agree	Strongly disagree
The presentation of the projected treatment pathways appeals to me.	◯	◯	◯	◯	◯
A medical weighing between the treatment pathways is important.	◯	◯	◯	◯	◯
The affinity score helps me make decisions.	◯	◯	◯	◯	◯
The system makes it easier for me to discuss options with discuss.	◯	◯	◯	◯	◯
The system makes it easier for me to discuss options with patients	◯	◯	◯	◯	◯
The system allows me to keep up with the latest research.	◯	◯	◯	◯	◯

Would you use the system?

☐ No, reject such applications in principle
☐ No, the presented version does not meet my requirements
☐ Yes, for patients with special forms (e.g. nail psoriasis)
☐ Yes, for multimorbid or pregnant patients
☐ Yes, for patients who are eligible for systemic therapies
☐ Yes, if health insurers grant separate remuneration for this purpose

Have you used similar applications before?

◯ Yes ◯ No

What were the applications involved?

____

What do you see as barriers to future use of the therapy recommendation system?

☐ Lack of confidence in correct operation
☐ Too much time required
☐ Designations not intuitively understandable
☐ No added value to the previous way of working
☐ Unclear how recommendation comes about
☐ Relevant information is missing
☐ Other reasons

What information are you missing?

____

Other reasons

____

What design ideas do you have regarding the therapy recommendation system?

Demographic issues

How old are you?

____
(Years)

Which gender do you have?

○ Male ○ Female ○ Diverse

What medical specialty do you work in?

○ Dermatology ○ Rheumatology ○ Pediatrics ○ Other

What is the field of expertise?

____

In which facility do you work?

○ Individual practice ○ Joint practice
○ Medical care center (employed) ○ Employed in a hospital
○ Other

What other facility are you in?

____

What position do you hold in the clinic?

○ Assistant physician ○ Specialist ○ Senior physician ○ Chief physician

How many psoriasis patients do you treat in a typical work week?

○ often none ○ 1-4 patients ○ 5-9 patients ○ 10 and more patients

How much professional experience (rounded to whole years) in dermatology do you have?

____
(Years)

Which medical specialization(s) do you have (multiple answers possible)?

☐ Allergology ☐ Andrology ☐ Dermatohistopathology ☐ Dermatoonkology ☐ Conservative Dermatology ☐ Operative Dermatology ☐ Sonography - Phlebology ☐ Venerology
