# Supplementary material for: Acceptance of a digital therapy recommender system for psoriasis
Source: BMC Med Inform Decis Mak. 2023 Aug 4;23:150. doi: 10.1186/s12911-023-02246-9 (PMC10401871; doi:10.1186/s12911-023-02246-9)
Supplement: Supplementary file 2 — Supplementary Material 2 [file 12911_2023_2246_MOESM2_ESM.pdf]

record_id	usability_timestamp	de_0	sus1	sus2	sus3	sus4	sus5	sus6	sus7	sus8	sus9	sus10	derma1	derma2	derma3	derma5	derma4	derma6	de_1__1	de_1__2	de_1__3	de_1__5	de_1__4	de_1__6	de_3__1	de_3__2	de_3__5	de_3__6	de_3__7	de_3__8	de_3__9	de_2	de_4	de_9	de_10	de_13	de_12	Sector	patient count	SUS10
U1	13.01.2020 16:48		Strongly agree	Rather not agree	Strongly agree	Strongly disagree	Strongly agree	Rather not agree	I rather agree	Rather not agree	Strongly agree	Rather not agree	Strongly agree	Strongly agree	I rather agree	Strongly agree	part-part	Strongly agree	Unchecked	Unchecked	Unchecked	Checked	Checked	Unchecked	Unchecked	Checked	Unchecked	Unchecked	Unchecked	Checked	Unchecked	29	Female	Employed in a hospital		10 and more patients	4	inpatient	2	8,75
U2	20.01.2020 16:53	Katja Müller	I rather agree	Strongly disagree	I rather agree	part-part	I rather agree	Strongly disagree	Strongly agree	Strongly disagree	Strongly agree	Rather not agree	I rather agree	Strongly agree	I rather agree	I rather agree	I rather agree	Strongly agree	Unchecked	Unchecked	Checked	Checked	Checked	Checked	Checked	Unchecked	Unchecked	Unchecked	Unchecked	Unchecked	Unchecked	27	Female	Employed in a hospital		10 and more patients	1	inpatient	2	8,5
U31	07.02.2020 12:26	Uwe Ostermann	I rather agree	Rather not agree	I rather agree	Strongly disagree	Strongly agree	Rather not agree	I rather agree	Rather not agree	I rather agree	Rather not agree	I rather agree	I rather agree	I rather agree	I rather agree	I rather agree	I rather agree	Unchecked	Unchecked	Unchecked	Unchecked	Checked	Unchecked	Unchecked	Unchecked	Unchecked	Unchecked	Unchecked	Unchecked	Unchecked	61	Female	Joint practice		10 and more patients	30	outpatient	2	8
U3	07.02.2020 12:31	Christine Eriksson	I rather agree	Strongly disagree	Strongly agree	Rather not agree	Strongly agree	Strongly disagree	Strongly agree	Strongly disagree	Strongly agree	Strongly disagree	Strongly agree	Strongly agree	Strongly agree	Strongly agree	Strongly agree	Strongly agree	Unchecked	Unchecked	Unchecked	Checked	Unchecked	Unchecked	Unchecked	Checked	Unchecked	Unchecked	Unchecked	Unchecked	Unchecked	29	Female	Employed in a hospital		10 and more patients	1	inpatient	2	9,5
U91	07.02.2020 12:33	Christine Eriksson	I rather agree	Rather not agree	part-part	Rather not agree	part-part	Rather not agree	I rather agree	Rather not agree	I rather agree	Strongly disagree	I rather agree	Strongly agree	I rather agree	I rather agree	part-part	I rather agree	Unchecked	Unchecked	Unchecked	Unchecked	Checked	Unchecked	Unchecked	Unchecked	Unchecked	Unchecked	Unchecked	Unchecked	Checked	64	Male	Employed in a hospital		10 and more patients	29	inpatient	2	7,25
U4	07.02.2020 12:47	Katja Müller	Strongly agree	I rather agree	I rather agree	I rather agree	I rather agree	Rather not agree	I rather agree	Rather not agree	I rather agree	Rather not agree	I rather agree	I rather agree	part-part	I rather agree	part-part	I rather agree	Unchecked	Unchecked	Checked	Checked	Checked	Checked	Unchecked	Unchecked	Unchecked	Unchecked	Checked	Unchecked	Unchecked	45	Female	Medical care center (employed)		5-9 patients	20	outpatient	2	6,75
U92	07.02.2020 12:48	Christine Eriksson	Strongly agree	Strongly disagree	I rather agree	Rather not agree	I rather agree	Rather not agree	I rather agree	Strongly disagree	I rather agree	Strongly disagree	I rather agree	I rather agree	I rather agree	Strongly agree	part-part	part-part	Unchecked	Unchecked	Unchecked	Unchecked	Checked	Unchecked	Unchecked	Unchecked	Unchecked	Unchecked	Unchecked	Unchecked	Checked	35	Female	Employed in a hospital		5-9 patients	5	inpatient	2	8,5
U32	07.02.2020 12:48	Uwe Ostermann	I rather agree	part-part	Rather not agree	Strongly disagree	Rather not agree	Strongly disagree	part-part	Strongly agree	Strongly disagree	Strongly agree	Strongly disagree	Strongly agree	part-part	Strongly disagree	Strongly disagree	Strongly agree	Unchecked	Unchecked	Checked	Unchecked	Unchecked	Unchecked	Checked	Checked	Unchecked	Unchecked	Checked	Unchecked	Unchecked	29	Male							4,25
U93	07.02.2020 12:59	Christine Eriksson	I rather agree	Rather not agree	I rather agree	Strongly disagree	part-part	part-part	Strongly agree	Strongly disagree	Strongly agree	Strongly disagree	part-part	I rather agree	I rather agree	I rather agree	part-part	Rather not agree	Unchecked	Unchecked	Checked	Checked	Checked	Unchecked	Unchecked	Checked	Unchecked	Checked	Checked	Unchecked	Unchecked	63	Male	Other	BAG	10 and more patients	26	outpatient	2	8,25
U5	07.02.2020 13:00	Uwe Ostermann	I rather agree	Rather not agree	I rather agree	Strongly disagree	I rather agree	part-part	I rather agree	Rather not agree	I rather agree	Strongly disagree	part-part	I rather agree	I rather agree	I rather agree	I rather agree	Strongly agree	Unchecked	Unchecked	Checked	Checked	Checked	Checked	Unchecked	Unchecked	Unchecked	Unchecked	Unchecked	Unchecked	Unchecked	27	Female	Employed in a hospital		often none	1	inpatient	1	7,75
U33	07.02.2020 13:09	Uwe Ostermann	Strongly agree	Strongly disagree	Strongly agree	Rather not agree	Strongly agree	Strongly disagree	Strongly agree	I rather agree	I rather agree	Strongly disagree	Strongly agree	Strongly agree	I rather agree	Strongly agree	Strongly agree	I rather agree	Unchecked	Unchecked	Unchecked	Unchecked	Checked	Unchecked	Unchecked	Unchecked	Unchecked	Unchecked	Unchecked	Unchecked	Unchecked	59	Male	Individual practice		5-9 patients	25	outpatient	2	8,75
U6	07.02.2020 13:15	Christine Eriksson	I rather agree	Rather not agree	I rather agree	Strongly disagree	Strongly disagree	part-part	I rather agree	Rather not agree	Strongly agree	Strongly disagree	I rather agree	Strongly agree	Rather not agree	I rather agree	I rather agree	part-part	Unchecked	Unchecked	Unchecked	Checked	Checked	Checked	Checked	Unchecked	Unchecked	Unchecked	Checked	Checked	Checked	30	Male	Employed in a hospital		5-9 patients	4	inpatient	2	7,25
U94	07.02.2020 13:24	Christine Eriksson	part-part	Strongly disagree	Rather not agree	Strongly agree	I rather agree	part-part	I rather agree	Rather not agree	Strongly disagree	Rather not agree	part-part	Strongly agree	I rather agree	I rather agree	part-part	I rather agree	Unchecked	Unchecked	Unchecked	Checked	Checked	Checked	Unchecked	Unchecked	Unchecked	Unchecked	Checked	Unchecked	Unchecked	46	Female	Joint practice		10 and more patients	20	outpatient	2	5,25
U7	07.02.2020 13:39	Katja Müller	Strongly agree	Rather not agree	Strongly agree	I rather agree	I rather agree	Rather not agree	part-part	Rather not agree	I rather agree	Strongly disagree	I rather agree	Strongly agree	Strongly agree	Strongly agree	Strongly agree	part-part	Unchecked	Unchecked	Unchecked	Unchecked	Checked	Unchecked	Unchecked	Checked	Unchecked	Unchecked	Unchecked	Unchecked	Unchecked	46	Female	Employed in a hospital		1-4 patients	15	inpatient	1	7,5
U95	07.02.2020 13:41	Christine Eriksson	I rather agree	Rather not agree	I rather agree	Strongly disagree	I rather agree	Rather not agree	Strongly agree	Rather not agree	I rather agree	Rather not agree	I rather agree	Strongly agree	I rather agree	part-part	part-part	Strongly agree	Unchecked	Unchecked	Unchecked	Checked	Checked	Unchecked	Checked	Unchecked	Unchecked	Unchecked	Unchecked	Unchecked	Unchecked	33	Female	Employed in a hospital		1-4 patients	1	inpatient	1	8
U8	07.02.2020 13:57	Uwe Ostermann	I rather agree	Rather not agree	part-part	I rather agree	part-part	part-part	I rather agree	part-part	I rather agree	Rather not agree	Strongly agree	I rather agree	I rather agree	I rather agree	Strongly agree	I rather agree	Unchecked	Unchecked	Unchecked	Unchecked	Checked	Unchecked	Unchecked	Unchecked	Unchecked	Unchecked	Unchecked	Unchecked	Unchecked	60	Male	Joint practice		10 and more patients		outpatient	2	6
U9	07.02.2020 14:36	Christine Eriksson	part-part	Rather not agree		I rather agree	I rather agree	I rather agree	I rather agree	Rather not agree	I rather agree	I rather agree	I rather agree	I rather agree	Rather not agree	part-part	I rather agree	I rather agree	Unchecked	Unchecked	Unchecked	Unchecked	Checked	Unchecked	Unchecked	Unchecked	Unchecked	Unchecked	Checked	Unchecked	Unchecked		Male	Individual practice		10 and more patients		outpatient	2	
U61	07.02.2020 14:52	Katja Müller	I rather agree	Strongly disagree	I rather agree	Strongly disagree	I rather agree	Rather not agree	Strongly agree	Rather not agree	Strongly agree	Strongly disagree	I rather agree	Strongly agree	I rather agree	Strongly agree	Strongly agree	Strongly agree	Unchecked	Unchecked	Checked	Checked	Checked	Unchecked	Unchecked	Unchecked	Unchecked	Unchecked	Unchecked	Unchecked	Unchecked	30	Female	Employed in a hospital		1-4 patients	2	inpatient	1	8,75
U34	07.02.2020 14:54	Uwe Ostermann	I rather agree	Rather not agree	I rather agree	Rather not agree	I rather agree	Rather not agree	I rather agree	Rather not agree	part-part	Rather not agree	I rather agree	Strongly agree	I rather agree	I rather agree	I rather agree	I rather agree	Unchecked	Unchecked	Unchecked	Unchecked	Checked	Unchecked	Unchecked	Unchecked	Unchecked	Checked	Unchecked	Unchecked	Unchecked	32	Female	Employed in a hospital		1-4 patients	1	inpatient	1	7,25
U10	07.02.2020 14:56	Katja Müller	I rather agree	part-part	I rather agree	Rather not agree	I rather agree	part-part	I rather agree	Rather not agree	part-part	Rather not agree	part-part	I rather agree	I rather agree	I rather agree	part-part	I rather agree	Unchecked	Unchecked	Checked	Checked	Checked	Unchecked	Unchecked	Unchecked	Unchecked	Unchecked	Unchecked	Unchecked	Unchecked	45	Female	Individual practice		10 and more patients	13	outpatient	2	6,75
U97	07.02.2020 15:03	Christine Eriksson	I rather agree	part-part	Rather not agree	Rather not agree	I rather agree	part-part	Rather not agree	Rather not agree	part-part	Strongly disagree	I rather agree	I rather agree	part-part	I rather agree	I rather agree	I rather agree	Unchecked	Unchecked	Unchecked	Unchecked	Checked	Unchecked	Unchecked	Checked	Unchecked	Unchecked	Unchecked	Unchecked	Checked		Female	Individual practice		10 and more patients	10	outpatient	2	6
U35	07.02.2020 15:03	Uwe Ostermann	I rather agree	part-part	I rather agree	Strongly disagree	Strongly agree	Rather not agree	Strongly agree	Strongly disagree	Strongly agree	Strongly disagree	I rather agree	Strongly agree	I rather agree	part-part	I rather agree	Strongly agree	Unchecked	Unchecked	Unchecked	Unchecked	Checked	Unchecked	Unchecked	Unchecked	Unchecked	Unchecked	Checked	Unchecked	Unchecked	59	Female	Individual practice		10 and more patients	30	outpatient	2	8,75
U62	07.02.2020 15:15	Katja Müller	part-part	Rather not agree	I rather agree	Rather not agree	I rather agree	part-part	I rather agree	I rather agree	I rather agree	Rather not agree	I rather agree	I rather agree	I rather agree	I rather agree	part-part	I rather agree	Unchecked	Unchecked	Unchecked	Checked	Unchecked	Unchecked	Unchecked	Checked	Unchecked	Unchecked	Unchecked	Unchecked	Unchecked	31	Female	Employed in a hospital		1-4 patients	1	inpatient	1	6,5
U11	07.02.2020 15:19	Uwe Ostermann	part-part	part-part		Strongly agree	I rather agree	part-part	Rather not agree	Strongly disagree	Rather not agree	Rather not agree	part-part	Strongly agree	part-part	Strongly disagree	Strongly disagree	Rather not agree	Unchecked	Unchecked	Unchecked	Unchecked	Unchecked	Checked	Checked	Checked	Unchecked	Unchecked	Checked	Unchecked	Unchecked	52	Male	Individual practice		10 and more patients	20	outpatient	2	
U98	07.02.2020 15:26	Christine Eriksson	part-part	Rather not agree	I rather agree	I rather agree	I rather agree	Rather not agree	part-part	Rather not agree	I rather agree	Strongly disagree	I rather agree	Strongly agree	part-part	I rather agree	part-part	Strongly disagree	Unchecked	Unchecked	Checked	Unchecked	Checked	Unchecked	Unchecked	Checked	Unchecked	Unchecked	Checked	Unchecked	Unchecked	34	Female	Employed in a hospital		10 and more patients	3	inpatient	2	6,75
U12	07.02.2020 15:35	Christine Eriksson	I rather agree	Rather not agree	I rather agree	Rather not agree	I rather agree	part-part	I rather agree	Strongly disagree	Strongly agree	Strongly disagree	I rather agree	Strongly agree	I rather agree	I rather agree	I rather agree	I rather agree	Unchecked	Unchecked	Checked	Checked	Checked	Checked	Unchecked	Unchecked	Unchecked	Unchecked	Unchecked	Unchecked	Unchecked	63	Female	Individual practice		10 and more patients	38	outpatient	2	8
U99	07.02.2020 15:56	Christine Eriksson	part-part	Rather not agree	I rather agree	Strongly disagree	I rather agree	Rather not agree	Strongly agree	Strongly disagree	Strongly agree	Strongly disagree	I rather agree	Strongly agree	I rather agree	I rather agree	part-part	part-part	Unchecked	Unchecked	Checked	Checked	Unchecked	Unchecked	Unchecked	Checked	Unchecked	Checked	Unchecked	Unchecked	Unchecked	42	Female	Individual practice		10 and more patients	13	outpatient	2	8,5
U13	07.02.2020 15:59	Katja Müller	I rather agree	Strongly disagree	Strongly agree	Strongly disagree	part-part	part-part	Strongly agree	Strongly disagree	Strongly agree	Strongly disagree	I rather agree	Strongly agree	Strongly agree	Strongly agree	I rather agree	I rather agree	Unchecked	Unchecked	Checked	Checked	Checked	Unchecked	Unchecked	Checked	Checked	Unchecked	Checked	Checked	Unchecked	39	Female	Individual practice		10 and more patients		outpatient	2	8,75
U100	07.02.2020 16:17	Christine Eriksson	Strongly agree	Strongly disagree	Strongly agree	Rather not agree	I rather agree	Rather not agree	Strongly agree	Strongly disagree	Strongly agree	Strongly disagree	Strongly agree	Strongly agree	Strongly agree	Strongly agree	Strongly agree	Strongly agree	Unchecked	Unchecked	Checked	Checked	Checked	Unchecked	Unchecked	Unchecked	Unchecked	Unchecked	Unchecked	Unchecked	Unchecked	64	Female	Joint practice		10 and more patients	35	outpatient	2	9,25
U63	07.02.2020 16:33	Uwe Ostermann	I rather agree	Strongly disagree	I rather agree	part-part	I rather agree	Rather not agree	I rather agree	Rather not agree	I rather agree	part-part	I rather agree	I rather agree	I rather agree	I rather agree	part-part	I rather agree	Unchecked	Unchecked	Unchecked	Unchecked	Checked	Unchecked	Unchecked	Unchecked	Unchecked	Checked	Checked	Unchecked	Unchecked	27	Female	Other		1-4 patients		outpatient	1	7,25
U101	07.02.2020 16:34		Strongly agree	Strongly disagree	I rather agree	Strongly agree	I rather agree	Strongly disagree	I rather agree	Rather not agree	Rather not agree	part-part	I rather agree	Strongly agree	I rather agree	I rather agree	part-part	I rather agree	Unchecked	Unchecked	Checked	Checked	Checked	Checked	Unchecked	Unchecked	Unchecked	Unchecked	Unchecked	Unchecked	Unchecked	59	Female	Individual practice		10 and more patients	34	outpatient	2	6,75
U14	07.02.2020 16:35	Uwe Ostermann	I rather agree	Rather not agree	I rather agree	Rather not agree	I rather agree	Rather not agree	I rather agree	Strongly disagree	I rather agree	Rather not agree	I rather agree	I rather agree	I rather agree	I rather agree	I rather agree	I rather agree	Unchecked	Unchecked	Unchecked	Unchecked	Checked	Unchecked	Unchecked	Unchecked	Unchecked	Unchecked	Unchecked	Unchecked	Unchecked	46	Male	Joint practice		5-9 patients	19	outpatient	2	7,75
U15	07.02.2020 16:46	Christine Eriksson	I rather agree	Strongly disagree	I rather agree	Rather not agree	I rather agree	Strongly disagree	Strongly agree	Rather not agree	I rather agree	Rather not agree	I rather agree	Strongly agree	I rather agree	I rather agree	part-part	I rather agree	Unchecked	Unchecked	Unchecked	Unchecked	Checked	Unchecked	Unchecked	Checked	Unchecked	Unchecked	Unchecked	Unchecked	Unchecked	28	Female	Employed in a hospital		often none	3	inpatient	1	8,25

U102	07.02.2020 16:50	Christine Eriksson	I rather agree	Rather not agree	I rather agree	part-part	I rather agree	Rather not agree	part-part	Rather not agree	I rather agree	Rather not agree	I rather agree	I rather agree	I rather agree	I rather agree	I rather agree	I rather agree	Unchecked	Unchecked	Checked	Checked	Checked	Unchecked	Checked	Unchecked	Unchecked	Unchecked	Unchecked	Unchecked	Unchecked	34	Female	Employed in a hospital		often none	7	inpatient	1	7
U38	07.02.2020 17:09	Katja Müller	I rather agree	Strongly disagree	I rather agree	Strongly disagree	I rather agree	Rather not agree	I rather agree	Strongly disagree	I rather agree	Strongly disagree	part-part	Strongly agree	part-part	I rather agree	I rather agree	I rather agree	Unchecked	Unchecked	Unchecked	Unchecked	Checked	Checked	Unchecked	Checked	Unchecked	Unchecked	Checked	Unchecked	Unchecked	31	Male	Employed in a hospital		5-9 patients	4	inpatient	2	8,5
U104	07.02.2020 17:11	Christine Eriksson	Strongly agree	Rather not agree	I rather agree	Rather not agree	I rather agree	Rather not agree	I rather agree	Rather not agree	I rather agree	Rather not agree	I rather agree	I rather agree	I rather agree	Strongly agree	I rather agree	part-part	Unchecked	Unchecked	Unchecked	Checked	Checked	Unchecked	Unchecked	Checked	Unchecked	Unchecked	Unchecked	Unchecked	Unchecked	29	Female	Employed in a hospital		1-4 patients	1	inpatient	1	7,75
U16	07.02.2020 17:11	Katja Müller	I rather agree	Rather not agree	I rather agree	Strongly disagree	Rather not agree	Rather not agree	I rather agree	Rather not agree	I rather agree	Rather not agree	I rather agree	Strongly agree	Strongly agree	Strongly agree	Strongly agree	Strongly agree	Unchecked	Unchecked	Unchecked	Checked	Unchecked	Unchecked	Unchecked	Checked	Unchecked	Unchecked	Unchecked	Unchecked	Unchecked	30	Female	Employed in a hospital		10 and more patients	4	inpatient	2	7,25
U17	07.02.2020 17:37	Uwe Ostermann	I rather agree	Rather not agree	I rather agree	I rather agree	part-part	part-part	I rather agree	Rather not agree	I rather agree	I rather agree	I rather agree	Strongly agree	Strongly agree	I rather agree	part-part	I rather agree	Checked	Unchecked	Unchecked	Unchecked	Unchecked	Unchecked	Checked	Checked	Unchecked	Unchecked	Unchecked	Checked	Unchecked	45	Female	Medical care center (employed)		10 and more patients	2	outpatient	2	6
U18	07.02.2020 18:27	Christine Eriksson	I rather agree	Rather not agree	I rather agree	Rather not agree	I rather agree	Rather not agree	I rather agree	Rather not agree	I rather agree	I rather agree	I rather agree	I rather agree	I rather agree	I rather agree	Rather not agree	Rather not agree	Unchecked	Unchecked	Checked	Checked	Checked	Unchecked	Unchecked	Checked	Unchecked	Unchecked	Unchecked	Unchecked	Unchecked	38	Female	Medical care center (employed)		10 and more patients	15	outpatient	2	7
U19	07.02.2020 18:55	Katja Müller	I rather agree	Rather not agree	I rather agree	Rather not agree	I rather agree	Rather not agree	part-part	Rather not agree	I rather agree	Rather not agree	I rather agree	I rather agree	I rather agree	I rather agree	I rather agree	Rather not agree	Unchecked	Unchecked	Checked	Checked	Unchecked	Unchecked	Unchecked	Checked	Unchecked	Unchecked	Unchecked	Unchecked	Unchecked	28	Male	Employed in a hospital		1-4 patients		inpatient	1	7,25
U20	07.02.2020 19:10	Uwe Ostermann	part-part	Rather not agree	Strongly agree	Strongly disagree	Strongly agree	Strongly disagree	Strongly agree	Strongly disagree	Strongly agree	Strongly disagree	Strongly agree	Strongly agree	part-part	I rather agree	I rather agree	Strongly agree	Unchecked	Unchecked	Checked	Checked	Checked	Checked	Unchecked	Checked	Unchecked	Unchecked	Unchecked	Unchecked	Unchecked	29	Female	Employed in a hospital		1-4 patients	3	inpatient	1	9,25
U105	08.02.2020 10:06	Christine Eriksson	I rather agree	I rather agree	Strongly agree	I rather agree	I rather agree	part-part	I rather agree	Strongly agree	I rather agree	I rather agree	I rather agree	Strongly agree	Strongly agree	Strongly agree	Strongly agree	Strongly agree	Unchecked	Unchecked	Unchecked	Unchecked	Checked	Unchecked	Unchecked	Unchecked	Unchecked	Unchecked	Unchecked	Unchecked	Unchecked	56	Female	Individual practice		10 and more patients	30	outpatient	2	5,25
U39	08.02.2020 10:07	Uwe Ostermann	I rather agree	Rather not agree	I rather agree	Strongly disagree	Strongly agree	Strongly disagree	I rather agree	Strongly disagree	Strongly agree	Strongly disagree	Strongly agree	Strongly agree	I rather agree	Strongly agree	I rather agree	Strongly agree	Unchecked	Unchecked	Unchecked	Unchecked	Checked	Unchecked	Unchecked	Checked	Unchecked	Unchecked	Unchecked	Unchecked	Unchecked	43	Female	Joint practice		5-9 patients	17	outpatient	2	9
U21	08.02.2020 10:09	Christine Eriksson	I rather agree	Rather not agree	I rather agree	part-part	part-part	part-part	I rather agree	Rather not agree	part-part	part-part	I rather agree	I rather agree	part-part	I rather agree	I rather agree	I rather agree	Unchecked	Unchecked	Unchecked	Unchecked	Checked	Unchecked	Unchecked	Checked	Unchecked	Unchecked	Unchecked	Checked	Unchecked	29	Male	Individual practice		10 and more patients	4	outpatient	2	6,25
U37	08.02.2020 10:09	Katja Müller	part-part	part-part	I rather agree	part-part	I rather agree	Rather not agree	Strongly agree	part-part	part-part	Rather not agree	I rather agree	I rather agree	I rather agree	I rather agree	Rather not agree	Strongly agree	Unchecked	Unchecked	Checked	Checked	Unchecked	Unchecked	Unchecked	Checked	Unchecked	Unchecked	Unchecked	Unchecked	Checked	39	Female	Medical care center (employed)		5-9 patients	13	outpatient	2	6,5
U22	08.02.2020 10:21	Christine Eriksson	I rather agree	Rather not agree	Strongly agree	I rather agree	Strongly agree	Rather not agree	Strongly agree	Strongly disagree	I rather agree	Rather not agree	Rather not agree	Strongly agree	Strongly agree	Strongly agree	Strongly agree	Strongly agree	Unchecked	Unchecked	Checked	Checked	Checked	Unchecked	Checked	Unchecked	Unchecked	Unchecked	Checked	Unchecked	Unchecked	55	Female	Individual practice		10 and more patients	25	outpatient	2	8
U64	08.02.2020 10:22	Uwe Ostermann	Strongly agree	Rather not agree	I rather agree	Rather not agree	I rather agree	Rather not agree	I rather agree	Rather not agree	I rather agree	Rather not agree	I rather agree	I rather agree	I rather agree	I rather agree	part-part	part-part	Unchecked	Unchecked	Unchecked	Unchecked	Checked	Unchecked	Unchecked	Unchecked	Unchecked	Unchecked	Checked	Unchecked	Unchecked	29	Female	Employed in a hospital		1-4 patients	3	inpatient	1	7,75
U40	08.02.2020 10:22	Uwe Ostermann	I rather agree	Rather not agree	I rather agree	Strongly disagree	I rather agree	Rather not agree	I rather agree	Rather not agree		Strongly disagree	I rather agree	I rather agree	I rather agree	I rather agree	I rather agree	I rather agree	Unchecked	Unchecked	Unchecked	Unchecked	Checked	Unchecked	Unchecked	Unchecked	Unchecked	Unchecked	Unchecked	Unchecked	Unchecked		Female	Employed in a hospital		1-4 patients		inpatient	1	
U106	08.02.2020 10:24	Christine Eriksson	I rather agree	Strongly disagree	Strongly agree	Strongly disagree	I rather agree	part-part	I rather agree	Strongly disagree	I rather agree	Strongly disagree	I rather agree	Strongly agree	part-part	Rather not agree	Rather not agree	Strongly disagree	Unchecked	Unchecked	Unchecked	Unchecked	Checked	Unchecked	Unchecked	Unchecked	Unchecked	Checked	Unchecked	Unchecked	Unchecked	53	Female	Employed in a hospital		1-4 patients	20	inpatient	1	8,5
U23	08.02.2020 10:40	Katja Müller	Strongly agree	part-part	part-part	Strongly agree	Strongly agree	Rather not agree	I rather agree	Rather not agree	Strongly agree	Strongly disagree	Strongly agree	Strongly agree	part-part	Strongly agree	I rather agree	Strongly agree	Unchecked	Unchecked	Checked	Checked	Checked	Checked	Unchecked	Checked	Unchecked	Unchecked	Unchecked	Unchecked	Checked	31	Male	Other	Practice. employed	1-4 patients	1	outpatient	1	7,25
U107	08.02.2020 10:47	Christine Eriksson	Strongly agree	Strongly disagree	Strongly agree	part-part	Strongly agree	Strongly disagree	Strongly agree	Strongly disagree	I rather agree	Strongly disagree	Strongly agree	Strongly agree	Strongly agree	Strongly agree	part-part	part-part	Unchecked	Unchecked	Checked	Checked	Checked	Checked	Checked	Unchecked	Unchecked	Unchecked	Checked	Unchecked	Unchecked	29	Female	Employed in a hospital		often none	3	inpatient	1	9,25
U24	08.02.2020 10:48	Christine Eriksson	Strongly agree	Strongly disagree	Strongly agree	I rather agree	I rather agree	Strongly disagree	Strongly agree	Strongly disagree	I rather agree	Rather not agree	Strongly agree	Strongly agree	Strongly agree	Strongly agree	Strongly agree	Strongly agree	Unchecked	Unchecked	Checked	Checked	Checked	Unchecked	Unchecked	Unchecked	Unchecked	Unchecked	Unchecked	Unchecked	Unchecked	31	Female	Employed in a hospital		1-4 patients	4	inpatient	1	8,5
U108	08.02.2020 11:11	Uwe Ostermann	I rather agree	Rather not agree	I rather agree	Strongly disagree	I rather agree	Rather not agree	Strongly agree	Rather not agree	Strongly agree	Strongly disagree	Strongly agree	I rather agree	part-part	I rather agree	I rather agree	I rather agree	Unchecked	Unchecked	Checked	Checked	Unchecked	Checked	Unchecked	Checked	Unchecked	Unchecked	Unchecked	Unchecked	Unchecked	41	Female	Individual practice		5-9 patients	13	outpatient	2	8,5
U41	08.02.2020 11:11	Uwe Ostermann	part-part	Rather not agree	I rather agree	Rather not agree	I rather agree	Rather not agree	I rather agree	Rather not agree	part-part	Rather not agree	I rather agree	Strongly agree	I rather agree	Strongly agree	I rather agree	I rather agree	Unchecked	Unchecked	Unchecked	Unchecked	Checked	Checked	Checked	Checked	Unchecked	Unchecked	Unchecked	Unchecked	Unchecked	46	Female	Individual practice		10 and more patients	20	outpatient	2	7
U25	08.02.2020 11:14	Katja Müller	Strongly agree	Strongly disagree	Strongly agree	part-part	I rather agree	Strongly disagree	I rather agree	Strongly disagree	I rather agree	I rather agree	Strongly agree	Strongly agree	I rather agree	I rather agree	Strongly agree	I rather agree	Unchecked	Unchecked	Unchecked	Checked	Checked	Checked	Unchecked	Checked	Unchecked	Unchecked	Unchecked	Unchecked	Unchecked	34	Female	Employed in a hospital		10 and more patients	6	inpatient	2	8
U26	08.02.2020 11:34	Uwe Ostermann	I rather agree	Strongly disagree	Strongly agree	part-part	I rather agree	Strongly disagree	I rather agree	Rather not agree	part-part	I rather agree	I rather agree	Strongly agree	I rather agree	Strongly agree	Strongly agree	Strongly agree	Unchecked	Unchecked	Checked	Checked	Checked	Checked	Unchecked	Unchecked	Unchecked	Unchecked	Unchecked	Unchecked	Unchecked	78	Female	Individual practice		10 and more patients	55	outpatient	2	7,25
U109	08.02.2020 11:34	Christine Eriksson	Rather not agree	I rather agree	Rather not agree	Strongly disagree	part-part	Strongly agree	I rather agree	I rather agree	Rather not agree		Strongly disagree	part-part	Strongly disagree	Strongly disagree	Strongly disagree	Strongly disagree	Unchecked	Checked	Unchecked	Unchecked	Unchecked	Unchecked	Unchecked	Checked	Unchecked	Checked	Unchecked	Unchecked	Unchecked	59	Female	Individual practice		10 and more patients	29	outpatient	2	
U27	08.02.2020 11:54	Uwe Ostermann	part-part	Rather not agree	Strongly disagree	Strongly agree	I rather agree	Strongly disagree	Rather not agree	part-part	Strongly disagree	Strongly agree	part-part	part-part	Rather not agree	Strongly disagree	part-part	I rather agree	Unchecked	Unchecked	Unchecked	Unchecked	Unchecked	Checked	Unchecked	Unchecked	Unchecked	Unchecked	Checked	Unchecked	Unchecked		Female	Medical care center (employed)		10 and more patients		outpatient	2	3,75
U110	08.02.2020 11:59	Katja Müller	I rather agree	Strongly disagree	Strongly agree	Strongly disagree	I rather agree	Strongly disagree	Strongly agree	Strongly disagree	I rather agree	Strongly disagree	I rather agree	Strongly agree	I rather agree	Strongly agree	Strongly agree	Strongly agree	Unchecked	Unchecked	Unchecked	Unchecked	Checked	Unchecked	Unchecked	Unchecked	Unchecked	Unchecked	Unchecked	Unchecked	Unchecked	36	Female	Employed in a hospital		10 and more patients	5	inpatient	2	9,25
U65	08.02.2020 12:02	Katja Müller	Strongly agree	Strongly disagree	I rather agree	Strongly disagree	I rather agree	Strongly disagree	I rather agree	Rather not agree	I rather agree	Strongly disagree	Strongly agree	Strongly agree	I rather agree	I rather agree	I rather agree	Strongly agree	Unchecked	Unchecked	Checked	Checked	Checked	Unchecked	Unchecked	Unchecked	Unchecked	Unchecked	Unchecked	Unchecked	Unchecked	32	Female	Employed in a hospital		often none	4	inpatient	1	8,75
U28	08.02.2020 12:39	Katja Müller	Rather not agree	Rather not agree	I rather agree	Rather not agree	I rather agree	part-part	I rather agree	Rather not agree	I rather agree	Rather not agree	I rather agree	Strongly agree	I rather agree	I rather agree	I rather agree	I rather agree	Unchecked	Unchecked	Checked	Unchecked	Unchecked	Unchecked	Unchecked	Unchecked	Unchecked	Checked	Unchecked	Unchecked	Unchecked	54	Male	Individual practice		10 and more patients	28	outpatient	2	6,75
U111	08.02.2020 12:40	Christine Eriksson	part-part	Strongly disagree	I rather agree	I rather agree	Strongly agree	Strongly agree	I rather agree	part-part	part-part	I rather agree	I rather agree	Strongly agree	I rather agree	part-part	I rather agree	Strongly agree	Unchecked	Unchecked	Unchecked	Unchecked	Checked	Checked	Unchecked	Checked	Checked	Unchecked	Unchecked	Unchecked	Unchecked	39	Female	Medical care center (employed)		1-4 patients	7	outpatient	1	5,5
U66	08.02.2020 13:00	Katja Müller	I rather agree	Rather not agree	I rather agree	I rather agree	I rather agree	Rather not agree	I rather agree	Rather not agree	I rather agree	Strongly disagree	part-part	part-part	I rather agree	I rather agree	I rather agree	I rather agree	Unchecked	Unchecked	Unchecked	Unchecked	Checked	Unchecked	Unchecked	Checked	Unchecked	Unchecked	Unchecked	Unchecked	Unchecked	27	Male	Employed in a hospital		1-4 patients	1	inpatient	1	7,25
U42	08.02.2020 13:00	Katja Müller	I rather agree	Strongly disagree	Strongly agree	Strongly disagree	Strongly agree	Strongly disagree	I rather agree	Strongly disagree	I rather agree	Strongly disagree	I rather agree	I rather agree	I rather agree	I rather agree	part-part	part-part	Unchecked	Unchecked	Unchecked	Unchecked	Checked	Unchecked	Unchecked	Unchecked	Unchecked	Unchecked	Unchecked	Unchecked	Unchecked	30	Male	Employed in a hospital		10 and more patients	3	inpatient	2	9,25
U29	08.02.2020 13:03	Uwe Ostermann	I rather agree	part-part	I rather agree	I rather agree	I rather agree	Rather not agree	I rather agree	Rather not agree	I rather agree	part-part	I rather agree	I rather agree	I rather agree	I rather agree	I rather agree	I rather agree	Unchecked	Unchecked	Checked	Checked	Checked	Checked	Unchecked	Unchecked	Unchecked	Unchecked	Unchecked	Unchecked	Unchecked	33	Female	Employed in a hospital		1-4 patients	3	inpatient	1	6,5
U30	08.02.2020 13:45	Katja Müller	Strongly agree	Rather not agree	I rather agree	Strongly disagree	I rather agree	I rather agree	Strongly agree	Strongly disagree	I rather agree	Strongly disagree	I rather agree	Strongly agree	I rather agree	I rather agree	I rather agree	part-part	Unchecked	Unchecked	Unchecked	Unchecked	Checked	Unchecked	Unchecked	Unchecked	Unchecked	Unchecked	Unchecked	Unchecked	Unchecked	59	Male	Individual practice		10 and more patients	25	outpatient	2	8,25
U113	08.02.2020 13:47	Christine Eriksson	Strongly agree	Strongly agree	Strongly agree	Strongly agree	Strongly agree	Strongly agree	Strongly agree	Rather not agree	I rather agree	Rather not agree	I rather agree	I rather agree	I rather agree	I rather agree	part-part	I rather agree	Unchecked	Unchecked	Unchecked	Unchecked	Checked	Unchecked	Unchecked	Checked	Unchecked	Unchecked	Unchecked	Unchecked	Unchecked	27	Female	Employed in a hospital		1-4 patients	1	inpatient	1	6,25

U114	08.02.2020 14:39	Christine Eriksson	I rather agree	Rather not agree	part-part	Rather not agree	Strongly agree	Strongly disagree	I rather agree	part-part	I rather agree	Rather not agree	Strongly agree	Strongly agree	Strongly agree	Strongly agree	Strongly agree	Strongly agree	Unchecked	Unchecked	Unchecked	Unchecked	Checked	Unchecked	Unchecked	Checked	Unchecked	Unchecked	Checked	Unchecked	Unchecked	59	Female	Individual practice	10 and more patients	32	outpatient	2	7,5
U60	08.02.2020 14:44	Katja Müller	Strongly agree	Strongly disagree	Strongly agree	Strongly disagree	Strongly agree	Strongly disagree	Strongly agree	Strongly disagree	I rather agree	Strongly disagree	Strongly agree	Strongly agree	Strongly agree	Strongly agree	Strongly agree	Strongly agree	Unchecked	Unchecked	Checked	Checked	Checked	Unchecked	Checked	Unchecked	Unchecked	Unchecked	Checked	Unchecked	Unchecked	49	Female	Individual practice	10 and more patients	20	outpatient	2	9,75
U36	12.03.2020 10:36	Katja Müller	I rather agree	Rather not agree	I rather agree	Strongly disagree	Strongly agree	Strongly disagree	Strongly agree	Strongly disagree	Strongly agree	Strongly disagree	Strongly agree	I rather agree	I rather agree	Strongly agree	I rather agree	I rather agree	Unchecked	Unchecked	Checked	Checked	Checked	Unchecked	Unchecked	Checked	Unchecked	Unchecked	Unchecked	Unchecked	Unchecked	29	Female	Employed in a hospital	1-4 patients	2	inpatient	1	9,25
U43	12.03.2020 14:16		Strongly agree	Strongly disagree	Strongly agree	Strongly disagree	Strongly agree	Strongly disagree	I rather agree	Strongly disagree	Strongly agree	Strongly disagree	Strongly agree	Strongly agree	Strongly agree	Strongly agree	Strongly agree	I rather agree	Unchecked	Unchecked	Checked	Checked	Checked	Unchecked	Unchecked	Unchecked	Unchecked	Unchecked	Checked	Unchecked	Unchecked	30	Female	Employed in a hospital	1-4 patients	5	inpatient	1	9,75
U44	12.03.2020 16:04	Uwe Ostermann	Strongly agree	Strongly disagree	Strongly agree	Rather not agree	Strongly agree	Rather not agree	Strongly agree	Strongly disagree	Strongly agree	Strongly disagree	Strongly agree	Strongly agree	Strongly agree	Strongly agree	I rather agree	part-part	Unchecked	Unchecked	Checked	Checked	Checked	Checked	Unchecked	Checked	Unchecked	Unchecked	Checked	Unchecked	Unchecked	34	Female	Employed in a hospital	often none	5	inpatient	1	9,5
U45	27.03.2020 09:46	Christine Eriksson	Strongly agree	Strongly disagree	Strongly agree	Strongly disagree	Strongly agree	Strongly disagree	Strongly agree	Strongly disagree	Strongly agree	Strongly disagree	Strongly agree	Strongly agree	I rather agree	I rather agree	Rather not agree	I rather agree	Unchecked	Unchecked	Unchecked	Unchecked	Checked	Unchecked	Unchecked	Checked	Unchecked	Unchecked	Unchecked	Unchecked	Unchecked	51	Male	Employed in a hospital	10 and more patients	21	inpatient	2	10
U47	02.04.2020 15:00	Uwe Ostermann	I rather agree	Strongly disagree	I rather agree	part-part	I rather agree	Rather not agree	Strongly agree	Strongly disagree	I rather agree	Strongly disagree	I rather agree	I rather agree	part-part	I rather agree	I rather agree	I rather agree	Unchecked	Unchecked	Unchecked	Unchecked	Checked	Unchecked	Unchecked	Checked	Unchecked	Unchecked	Unchecked	Unchecked	Unchecked	34	Female	Employed in a hospital	1-4 patients	5	inpatient	1	8,25
U49	16.06.2020 09:40	Uwe Ostermann	I rather agree	Strongly disagree	Strongly agree	Rather not agree	I rather agree	Strongly disagree	Strongly agree	Strongly disagree	I rather agree	Rather not agree	Strongly agree	Strongly agree	I rather agree	Strongly agree	I rather agree	I rather agree	Unchecked	Unchecked	Checked	Checked	Checked	Checked	Unchecked	Unchecked	Unchecked	Unchecked	Checked	Unchecked	Checked	32	Female	Employed in a hospital	10 and more patients	4	inpatient	2	8,75
U46	24.06.2020 14:48	Uwe Ostermann	I rather agree	Rather not agree	part-part	part-part	I rather agree	Rather not agree	I rather agree	part-part	part-part	Rather not agree	part-part	I rather agree		part-part	part-part	part-part	Unchecked	Unchecked	Checked	Unchecked	Unchecked	Unchecked	Unchecked	Checked	Checked	Unchecked	Unchecked	Unchecked	Unchecked	39	Female	Employed in a hospital	1-4 patients		inpatient	1	6,5

Variable	Label
record_id	Person identifier
usability_timestamp	time when questionair was sent to System
de_0	Name of Pseuo Patient the person looked at
sus1	I think that I would like to use this system frequently.
sus2	I found the system unnecessarily complex.
sus3	I thought the system was easy to use.
sus4	I think that I would need the support of a technical person to be able to use this system.
sus5	I found the various functions in this system were well integrated.
sus6	I thought there was too much inconsistency in this system.
sus7	I would imagine that most people would learn to use this system very quickly.
sus8	I found the system very cumbersome to use.
sus9	I found the system very cumbersome to use.
sus10	I needed to learn a lot of things before I could get going with this system.
derma1	The presentation of the projected treatment pathways appeals to me
derma2	A medical weighing between the treatment pathways is important.
derma3	The affinity score helps me make decisions.
derma5	The system makes it easier for me to discuss options with discuss.
derma4	The system makes it easier for me to discuss options with patients
derma6	The system allows me to keep up with the latest research.
de_1___1	No, reject such applications in principle
de_1___2	No, the presented version does not meet my requirements
de_1___3	Yes, for patients with special forms (e.g. nail psoriasis)
de_1___5	Yes, for multimorbid or pregnant patients

de_1___4	Yes, for patients who are eligible for systemic therapies
de_1___6	Yes, if health insurers grant separate remuneration for this purpose
de_3___1	Lack of confidence in correct operation
de_3___2	Too much time required
de_3___5	Designations not intuitively understandable
de_3___6	No added value to the previous way of working
de_3___7	Unclear how recommendation comes about
de_3___8	Relevant information is missing
de_3___9	Other reasons
de_2	Age in years
de_4	Sex
de_9	In which facility do you work?
de_10	Other facility
de_13	How many psoriasis patients do you treat in a typical work week?
de_12	How much professional experience (rounded to whole years) in dermatology do you have?
Sector	generated inpatient/outpatient sector
patientcount	generated patient count with psoriais in a week
SUS10	Score System usability scale per 10 units
